# Recent technical advances in proteomics

**DOI:** 10.12688/f1000research.16987.1

**Published:** 2019-03-29

**Authors:** John R. Yates

**Affiliations:** 1 Molecular Medicine and Neurobiology, Scripps Research, 0550 North Torrey Pines Road, SR302, La Jolla, CA, 92037, USA

**Keywords:** proteomics, mass spectrometry, spatial proteomics, interaction proteomics

## Abstract

Mass spectrometry is one of the key technologies of proteomics, and over the last decade important technical advances in mass spectrometry have driven an increased capability for proteomic discovery. In addition, new methods to capture important biological information have been developed to take advantage of improving proteomic tools.

Over the past 15 years, the study of genomics has frequently made headlines as DNA sequencing has uncovered genes that contribute to or cause disease. When DNA sequencing began to move into the clinic, it generated hope that more biology and disease buried in a genome would be uncovered. However, it soon became clear that the number of genome sequences needed to understand human biology would be far greater than expected. An understanding of multigenic human variability requires sequencing many different genomes to tease out the interplay of genes that create complex traits. Even after large-scale sequencing efforts, it has been difficult to measure susceptibility for multigenic diseases, leading to a concern about missing patterns of heritability for some diseases ^1, 2^. For example, mutations in genes that increase the risk of familial Alzheimer’s disease have been identified, but genetic risk for sporadic Alzheimer’s disease has been harder to establish ^3– 5^. Are there other genes that are important that are not so obvious or is there a larger role for environment than expected? We are beginning to understand that DNA sequence mutations and variations may impact their protein products in yet-unknown ways and this may change as a function of age and environment and therefore may play an important role in health and disease ^6^.

Genes encode proteins, which are complex molecules that catalyze reactions, transmit signals, and create cellular support structures that possess a three-dimensional structure organized in a spatial and temporal manner ^7, 8^. In many of the initial proteomic studies, the focus was to associate specific proteins with biological processes. Although this effort has been successful, it is important to understand that proteins do not necessarily have a single function or role in physiology (for example, pleiotropy) and thus one of the great challenges of proteomics is uncovering the diverse cellular functions and roles of proteins in cells. Further advances in proteomics have focused on the identification of post-translational modifications of proteins, protein–protein interactions, and the locations of proteins within cells ^9– 11^. Post-translational modifications of proteins are not encoded in the genome; instead, amino acid sequence signatures for modification sites may be obtusely encoded within the sequence of a protein. Modifications often regulate protein activity or function and thus play an important role in the regulation of processes. Tens of thousands of myriad modification sites have been identified by large-scale proteomic studies, and attempts to identify specific sites as regulators of biological processes are ongoing ^12^. The exquisite complexity of modifications is perfectly exemplified in histones, which have intricate patterns of modifications on the exposed tails of the proteins. The functions or activities of proteins are separated by spatial organization in organelles or subcellular compartments. Identifying proteins present in organelles (that is, mitochondria) helps to define the roles of proteins as well as potential functions that may be carried out in a compartment and this information can better define the functions of the organelles ^13^. Recent proteomic studies seek to determine the structures or folding of proteins on a large scale and *in vivo* ^14^. Advances in cryogenic electron microscopy (cryo-EM) have resulted in a tremendous increase in the number of difficult structures that have been determined for proteins ^15^. However, so far, these studies are performed mostly *in vitro*, so it is important to determine how these structures conform to those in cells. Mass spectrometry (MS) has been successfully employed for the analysis of native proteins and native protein complexes and now is being used to study whole cells in an attempt to measure the state of folding of proteins within complete proteomes ^14, 16^. To advance the capture of proteomic information, new instrumentation and methodologies are needed.

Developments in mass spectrometers over the last decade have been numerous, but there are some clear trends. The drive to increase confidence in the identification of peptides and post-translational modifications pushed the development of high-resolution and high-mass accuracy instruments, most notably Orbitrap and time-of-flight (TOF) mass analyzers ^17– 20^. Improvements in mass resolution in these instruments resulted in an increase in the mass range for effective analysis, precipitating greater interest in the “top down” proteomics which now could be performed without expensive high-field magnets previously required for ion cyclotron resonance MS of intact proteins ^21– 23^. Additionally, the emergence of biological therapeutics has fueled a greater need to characterize intact proteins to verify structure, sequence, and modifications ^24– 26^. Fragmentation of the amide bonds in intact proteins requires more robust methods than fragmentation of peptides to obtain sequence information ^27^. Two methods in particular—electron transfer dissociation (ETD) and ultraviolet photodissociation (UVPD)—have been used to achieve more efficient fragmentation of intact proteins, especially when used in combination ^28– 31^. These substantial improvements in MS capability have led to greatly improved prospects for top-down MS.

A common strategy to improve the performance of mass spectrometers has been to create hybrid instruments. A hybrid instrument uses different ion analyzers or separators to increase the capabilities of the mass spectrometer as a whole. For example, development of the triple quadrupole mass spectrometer led to big improvements in performance over a single quadrupole instrument by adding two other quadrupoles; one quadrupole was used to select m/z values, another was used as a collision cell, and the third quadrupole was used to perform more routine analysis of ions. A more recent hybrid instrument is the Orbitrap Fusion Lumos Tribrid mass spectrometer, which includes five different ion separation/storage devices ^32^. In the Orbitrap Fusion Lumos Tribrid, the quadrupole mass filter is used to select an m/z, and an ion routing multipole serves as a “traffic cop” to store and direct ions to either a linear ion trap for collision-induced dissociation or to the Orbitrap for high-resolution and high-mass accuracy measurements. The ion routing multipole can also be used for higher-energy collisional dissociation (HCD) ion fragmentation. The use of an ion storage device like the ion routing multipole device allows simultaneous experiments within the instrument which can increase the effective scan speed and consequently the number of tandem mass spectra collected for peptide ions. Thus, scan speed is increased in the Orbitrap Fusion Lumos Tribrid by using the available ions more effectively, and routine analysis of digested protein mixtures results in a larger number of peptide (and hence protein) identifications. ****

Over the last 20 years, there has been increasing interest in using ion mobility spectrometer (IMS) devices to add ion separation capabilities to mass spectrometers. IMS devices use high-pressure gas and constraining electric fields to separate ions based on features besides m/z, thus providing improved separation of molecules before the mass analyzer ^33, 34^. Hoaglund *et al*. used ion mobility separation in conjunction with a quadrupole TOF mass spectrometer to analyze peptide mixtures, and the success of this experiment triggered further interest in IMS devices as adjuncts to traditional mass analyzers ^35^. As a result, a variety of devices have emerged based on the ion mobility concept, including the traveling wave, which uses an electrical wave (and lower gas pressure) instead of a constant high voltage to drive ions through a gas ^36^. A trapped IMS (TIMS) device uses electric and radiofrequency fields to trap ions in a flowing gas ^37^. In the TIMS device, ion motion against the gas determines the resolution of the separation. The success of TIMS led to the development of parallel accumulation-serial fragmentation (PASEF), which is a mass selective release of peptide ions from the TIMS device for MS/MS ^38^. Combining these methods (TIMS/PASEF) provides another means to fractionate complex mixtures of ions to increase the number of tandem mass spectra of peptides collected and thus the number of protein identifications.

A different type of ion mobility, differential mobility spectrometer or field asymmetric IMS (FAIMS), has been used to create separation of ions ^39^. In this instrument, ions pass through a gas with an orthogonal field driving ions toward the wall of the cell. Based on the selection of the electric field, ions of a certain m/z will pass through to the outlet of the device. When coupled to a mass spectrometer, FAIMS can decrease the complexity of ions entering the mass spectrometer and can selectively pass through different sets of ions by systematically changing the electric field.

A very exciting development in the ion mobility field is a device called structures for lossless ion manipulations (SLIMs) ^40– 42^, which makes use of the traveling wave principle to move ions. SLIMs are fabricated from printed circuit board technology and thus are inexpensive to create and have great flexibility in design and construction. Features have been added to turn ions around corners and to effectively create very long path lengths that facilitate increased ion separations. Webb *et al*. have interfaced SLIM devices with TOF mass spectrometers to perform mass analysis ^43^. Because of the ease of construction and flexibility in design, these devices have enormous potential for creative separations, especially with the lossless nature of the ion manipulations.

High-resolution ion separations have enabled improved mass spectrometer performance for analysis of intact proteins with less sophisticated instruments which has increased interest in the application of top-down proteomics to biological problems. A common problem in protein analysis is measuring the proteoforms of a protein, which include all modifications and sequence variations present ^44^. It is important to identify all the modifications on a protein to determine how those modifications attenuate or alter the protein’s functions. Improvements in mass spectrometers and methodology are increasing the scale of intact protein analysis as well as the effective size of proteins that can be reasonably analyzed. Intact protein identification methods require fragment ion data at amide linkages throughout the backbone of the protein to both identify the proteins and more accurately localize modifications. As described above, the development of ETD and UVPD has enabled better fragmentation of proteins to more confidently assign modifications to sites within the protein.

Another rapidly improving method in MS is the analysis of native protein complexes, which has been advanced by Marcoux and Robinson to enable the characterization of membrane proteins and membrane protein complexes ^45^. A recent breakthrough in this area has been the use of surface-induced dissociation to fragment protein complexes ^46– 49^, which allows the user to direct a greater amount of the kinetic energy of the collision into the ion complex than if a gas-phase dissociation method is used. By varying the energy of the collisions, proteins on the outside of the native complex can be peeled away to reveal the organization of the complex. Skinner *et al*. have used non-denaturing separations of protein complexes in conjunction with analysis of the native complex and an MS3 approach for top-down identification of the individual components of the complex ^50, 51^. Using this strategy, the authors were able to determine proteoforms of the proteins in the complex. In these native protein analyses, UVPD has been used in conjunction with HCD to help fragment the protein. Snijder *et al*. elegantly used native MS to decipher the sequence of protein–protein interactions involved in the circadian rhythms of the Kai system in cyanobacterium ^16^.

The method used by Skinner *et al*. to separate protein complexes is based on a new strategy called protein correlation profiling (PCP) ^52, 53^. This method uses the co-elution of proteins under non-denaturing conditions as a measure of whether the proteins are in a complex together and is based on the theory that if proteins co-elute under different chromatographic conditions, there is a calculable probability that they are present together in a complex. This strategy may offer a means to more quickly measure the dynamics of protein complexes in systems undergoing some sort of perturbation such as disease or drug treatment. Cross-linking of complexes has been used to maintain the integrity of complexes that might involve membrane proteins, as they sometimes require buffers that might be denaturing to maintain solubility ^54^. Very large-scale studies have been performed using individual pull-downs of proteins to obtain protein complexes and these studies have provided the reference sets to establish the validity of methods such as PCP ^55, 56^. However, pull-down approaches are too time-consuming to be practical for the study of protein dynamics on a large scale. PCP may prove to be a good solution to the time constraints of studying large-scale protein complex dynamics, but more development of the method will be required.

The measurement of protein interactions using affinity pull-downs and MS has been a powerful method for the discovery of protein interactions, but for interactions to survive the enrichment process they needs to have a certain level of affinity to the interaction. “Spatial proteomics” is a recent method that determines proteins in the region around a bait without requiring a high level of affinity between interactors; rather, the method requires only that interactors be within a defined region. Two methods for spatial proteomics have been developed. One method employs an engineered version of ascorbic acid peroxidase (APEX) fused to proteins which produces a hydroxyl radical when hydrogen peroxide and phenoxy biotin are added and labels proteins within 30 angstroms of APEX ^13^. The biotinylated proteins then can be enriched for analysis. The BioID strategy uses the biotin ligase enzyme BirA to collect the same type of data ^57– 60^. In BioID, BirA ligase is added to a bait protein, and when biotin is fed to a cell, biotin is added to nearby lysine residues. APEX is much faster than BioID, which can require up to 24 hours to get sufficient labeling, allowing the BirA-labeled protein time to move around the cell if it is not fixed to a membrane. In both methods, good controls are required to differentiate signal from noise. Recently, Branon *et al*. engineered a version of the BirA protein that is able to sufficiently label proteins in 10 minutes in a method aptly named TurboID ^61^. A very clever use of APEX involved epitope tagging of CAS9 with the ascorbate enzyme ^62^. In this application, CAS9 guide RNAs are used to place the labeled CAS9 at a specific gene, where it labels proteins in the vicinity, including transcription factors and histones that subsequently can be enriched for identification and post-translational modification analysis. Spatial proteomics is drawing particular interest for determining the type of histone modifications that are present at a specific location in the genome. Spatial proteomic methods provide a strategy to supplement the type of information that might be derived from protein–protein interaction studies.

The use of biorthogonal chemistry has exploded over the last two decades as a means to label proteins or glycans within cells ^63^. Biorthogonal chemistry uses specific types of biomolecules that can be metabolized in cells or tissues and inserted into proteins or glycans. Generally, the molecule will have an affinity tag to allow enrichment of the modified proteins after insertion. A particularly interesting molecule is azidohomoalanine (AHA), which can be used by the endogenous Met t-RNA synthetase to incorporate AHA into proteins ^64^. AHA then can be reacted with biotin alkyne using a copper-catalyzed Huisgen 1,3-dipolar cycloaddition in aqueous solution ^64^, and the biotin-labeled proteins can be enriched using avidin. This has become a powerful method to introduce affinity labels into proteins. For instance, AHA has been used to determine the identity of newly synthesized proteins in response to perturbations and to identify proteins secreted by cells ^65^. A few strategies have been developed to quantitate proteins using AHA. The first method combines the introduction of AHA with stable isotope-labeled amino acids where heavy amino acids could be used for one state and light amino acids used in another state ^65^. A second strategy uses a heavy and light labeled version of biotin alkyne together with AHA as a means to quantitate ^66^. A third strategy uses a heavy and light version of AHA to provide quantitation ^67^. Each method has different advantages which pertain to when labels are introduced into the sample and the subsequent manipulations required. In the method that uses stable isotope-labeled amino acids, the introduction of labels is separated from the incorporation of AHA and thus quantitation errors introduced by sample handling inequities are minimized, although mixing errors of the heavy and light cells could occur. The process that uses heavy and light -biotin alkyne requires the introduction of the labels into two different samples which requires careful control over processes to avoid reaction or recovery errors. The last process of using a heavy and light AHA incorporates the label into proteins using the metabolic machinery of the cell and thus quantitation errors could stem from mixing or mass balance errors. An interesting application of AHA is for protein turnover measurements ^68^. The use of stable isotope-labeled amino acids for protein turnover measurements has been hampered by difficulty in distinguishing the isotope signatures in the midst of increasing background from normal signals. AHA can be pulsed into cells or animals followed by a chase of normal Met, and over time AHA can be recovered by affinity capture, providing a means to enrich very low levels that might be present at long time points.

Proteomic capability is constantly evolving as a result of technological and methodological advances in the field. Many of these advances come from improvements in MS technology that provides new capabilities and measurement improvements. Researchers then are able to leverage these advances into measurements of new features of biological systems and to improve the diagnosis of medical conditions.
